# Berberine Improves Irinotecan-Induced Intestinal Mucositis Without Impairing the Anti-colorectal Cancer Efficacy of Irinotecan by Inhibiting Bacterial β-glucuronidase

**DOI:** 10.3389/fphar.2021.774560

**Published:** 2021-11-02

**Authors:** Bei Yue, Ruiyang Gao, Cheng Lv, Zhilun Yu, Hao Wang, Xiaolong Geng, Zhengtao Wang, Wei Dou

**Affiliations:** The MOE Key Laboratory of Standardization of Chinese Medicines, Shanghai Key Laboratory of Compound Chinese Medicines, and the SATCM Key Laboratory of New Resources and Quality Evaluation of Chinese Medicines, Institute of Chinese Materia Medica, Shanghai University of Traditional Chinese Medicine (SHUTCM), Shanghai, China

**Keywords:** irinotecan, mucositis, colorectal cancer, β-glucuronidase, berberine

## Abstract

Irinotecan (CPT11), a broad-spectrum cytotoxic anticancer agent, induces a series of toxic side-effects. The most conspicuous side-effect is gastrointestinal mucositis, including nausea, vomiting, and diarrhea. A growing body of evidence indicates that bacteria β-glucuronidase (GUS), an enzyme expressed by intestinal microbiota, converts the inactive CPT11 metabolite SN38G to the active metabolite SN38 to ultimately induce intestinal mucositis. We sought to explore the potential efficacy and underlying mechanisms of berberine on CPT11-induced mucositis. Our study showed that berberine (50 mg/kg; i. g.) mitigated the CPT11-induced loss of mucosal architecture, ulceration, and neutrophil infiltration. Meanwhile, berberine improved mucosal barrier function by increasing the number of globlet cells, protecting trans-endothelial electrical resistance (TEER), reducing permeability and increasing tight junction proteins expression. LC-MS analysis showed that berberine decreased the content of SN38 in feces, which correlated with decreases in both GUS activity and GUS-producing bacteria. Further molecular docking and Lineweaver-Burk plots analyses suggested that berberine functions as a potential non-competitive inhibitor against GUS enzyme. Of note, berberine maintained the anti-tumor efficacy of CPT11 in a tumor xenograft model while abrogating the intestinal toxicity of CPT11. Overall, we identified for the first time the remission effects of berberine on intestinal mucositis induced by CPT11 without impairing the anti-colorectal cancer efficacy of CPT11 partially via inhibiting bacterial GUS enzyme.

## Introduction

The circumstances of chemotherapy-induced gastrointestinal toxicities involve inflammation and damage to mucosa, thus resulting in pain, nausea, vomiting, bloating, constipation, diarrhea and ulceration (Eng, 2009). As an inhibitor of topoisomerase I (TOP1), irinotecan (CPT11) is one of the major agents used in the treatment of metastatic or advanced solid tumors (e.g., gastric (Shintani et al., 2021), pancreatic (Glassman et al., 2018), ovarian (Mabuchi et al., 2019), colorectal (Reita et al., 2019), and other tumors (Bhamidipati et al., 2021)). However, about 80% of patients receiving standard dose of CPT11 chemotherapy exhibit diarrhea-associated intestinal mucositis (Gelibter et al., 2019). Moreover, the prevalence of severe diarrhea (defined as grade 3 or 4) caused by CPT11 occurs in 22–44% of patients (de Man et al., 2018). Previous studies have suggested that the occurrence of intestinal mucositis induced by diarrhea involves a series of pathological features including inflammatory response, tight junction defects, and mucosal barrier dysfunction (Boeing et al., 2020). Emerging studies have shown that genetic polymorphisms in drug metabolizing enzymes and drug transporters can affect irinotecan-induced toxicity (Atasilp et al., 2020; Riera et al., 2020).

As a prodrug, CPT11 is activated to become SN38 (a potent TOP1 inhibitor) by intestinal microbiota and exhibits repressive effects on cancer cells and rapidly dividing normal cells (Alimonti et al., 2004). Subsequently, SN38 is detoxified *via* the glucuronic acid reaction to form SN38G, an inactive compound that subsequently eliminated from the intestinal lumen (de Man et al., 2018). However, bacterial β-glucuronidase (GUS), which is distributed in the intestinal lumen, can regenerate SN38G to the active form SN38. Thus, the GUS enzyme prolongs the clearance time of CPT11 from the body and is therefore, considered to be a direct promotor of CPT11-induced intestinal mucositis (Kuhn, 1998). Cheng et al. has revealed that pharmacological inhibition of GUS by inhibitors alleviates the intestinal injury caused by CPT11 without diminishing the anti-tumor efficacy of CPT11 (Wallace et al., 2010; Cheng et al., 2015; Wallace et al., 2015). Correspondingly, another study has demonstrated that an old drug, amoxapine, enhances the anti-tumor efficacy of CPT11 in tumor-bearing mice while alleviating the toxic side-effects of CPT11 (Kong et al., 2014). The above pharmaceutical activity of amoxapine has been reported to be associated with the modulation of the GUS enzyme (Kong et al., 2014).

Intestinal mucositis has been investigated for more than 30 years. However, there is still no successful medical approach in clinical practice for preventing the inflammatory conditions (Santos Filho et al., 2016). Specifically, various herbs and natural compounds with anti-inflammatory properties have been studied as up-and-coming strategies for treating intestinal mucositis, including acteoside (Reinke et al., 2015), rutin (Boeing et al., 2020), *Bidens pilosa L* (de Avila et al., 2015), silymarin (Sasu et al., 2015), as well as herb prescriptions of PHY906 (Lam et al., 2014), Bu-Zhong-Yi-Qi (Gou et al., 2016), and Ge-Gen-Qin-Lian (Wu et al., 2019). Furthermore, some traditional plant-based medicines have been approved for the clinical management of chemotherapy-induced nausea and vomiting (Chen et al., 2016). Berberine [9,10-dimethoxy-5,6-dihydro-(1,3)dioxolo (4,5-g)isoquinolino (3,2-a)isoquinolin-7-ium] is a natural isoquinoline alkaloid mainly isolated from *Berberis aquifolium* Pursh, *Berberis vulgaris* L, and *Coptis chinensis* Franch (Cicero and Baggioni, 2016). Based on pharmacological studies, berberine exhibits a variety of biological activities including anti-inflammation, anti-oxidation, anti-cancer, regulation of lipid metabolism, and maintenance of energy balance (Ortiz et al., 2014; Wang et al., 2017). Previous studies revealed that berberine alleviates experimental colitis by maintaining intestinal barrier function, suppressing inflammatory responses, and regulating gut microbiota (Zhang et al., 2015; Li et al., 2020). However, the beneficial effects of berberine on intestinal mucositis induced by CPT11 chemotherapy and the underlying mechanisms have not been elucidated. In this study with a mouse model, we demonstrated that berberine alleviated intestinal mucositis induced by CPT11 partially via inhibition of bacterial GUS enzyme.

## Materials and Methods

### Materials

Berberine (C_20_H_18_NO_4_·Cl; CAS: 633-65-8; molecular weight: 371.81; high-performance liquid chromatography purity ≥98%) was purchased from Melone Pharmaceutical Co., Ltd (Dalian, China). Irinotecan (CPT11) (C_33_H_45_N_4_O_9_·Cl, CAS: 136572-09-3; molecular weight: 677.18) was purchased from Melone Pharmaceutical Co., Ltd (Dalian, China). β-glucuronidase (GUS, CAS: 9001-45-0, G7396-25kU) from *Escherichia coli* was obtained from Thermo Scientific Inc. (Waltham, MA, United States). NCM460 human intestinal epithelial cells, CT26 murine colon carcinoma cells and Caco2 human colorectal adenocarcinoma cells were purchased from the American Type Culture Collection (ATCC, Manassas, VA, United States). Roswell Park Memorial Institute (RPMI)-1640, Dulbecco’s modified Eagle’s medium (DMEM), 100 U/ml penicillin/streptomycin, and fetal bovine serum (FBS), were purchased from Gibco BRL (Grand Island, NY, United States). Diethylpyrocarbonate-treated water, dimethyl sulfoxide, paraformaldehyde, and diaminobenzidine were obtained from Sigma-Aldrich (Shanghai, China). We also acquired an enhanced chemiluminescence (ECL) detection kit from Millipore (Billerica, MA, United States). Rabbit antibodies raised against inducible nitric oxide synthase (iNOS) (#18985-1-AP), cyclooxygenase-2 (COX-2) (#12375-1-AP), and Claudin-7 (#10118-1-AP), were obtained from Proteintech Group (Chicago, IL, United States). A β-actin antibody (#4970) was obtained from Cell Signaling Technology (Danvers, MA, United States). Rabbit antibodies against zonula occludens 1 (ZO-1, #A11417), Claudin-1 (#A2196), and Occludin (#A2601), were obtained from ABclonal Technology (Wuhan, China). Trizol and a SuperScript II Reverse Transcriptase kit were purchased from Thermo Scientific Inc. (Waltham, MA, United States). SYBR Premix ExTaq Mix was purchased from Takara Biotechnology (Shiga, Japan).

### Animals

Both healthy female C57BL/6 mice (8-week-old, 20–22 g) and male BALB/c mice (6-week-old, 20–22 g) were purchased from the Shanghai Laboratory Animal Center in Shanghai University of Traditional Chinese Medicine. All mice were maintained in specific pathogen-free facility and kept under controlled conditions at a humidity of 60–70% and a temperature of 23–25°C in a 12 h light/dark cycle. The mice had access to autoclaved food and drinking water *ad libitum.* All animal experiments were conducted in accordance with the principles of the declaration and recommendations of the Animal Experimentation Ethics Committee at Shanghai University of Traditional Chinese Medicine (Animal Ethics Number: PZSHUTCM200828001 and PZSHUTCM200724006, respectively).

### Investigating CPT11-Induced Intestinal Mucositis and Antitumor Effects *in Vivo*

Experimental intestinal mucositis was induced in mice by the intraperitoneal injection of CPT11 (50 mg/kg). C57BL/6mice were randomly divided into the following groups (*n* = 6 mice per group): control group, CPT11 group, and CPT11 + Berberine group. Control mice were injected with vehicle (5% DMSO). Except for the control group, all other groups of mice were injected with CPT11 (50 mg/kg) for 14 days to induce intestinal mucositis. Mice in the CPT11 + Berberine group were treated daily with berberine (50 mg/kg/day) from day 1 to day 14. Berberine was dissolved in 0.5% methylcellulose and administered by oral gavage. Body weights were recorded daily, and mice were sacrificed under anesthesia after receiving the last gavage. Blood was collected from the eye orbits of each mouse. Subsequently, the entire colon was removed, and the total length was measured. Then, the feces were collected from the interior of the rectum. Distal colon tissues were collected for hematoxylin–eosin (H&E) staining and Alcian blue (AB)/Periodic Acid-Schiff stain (PAS) staining. The number of goblet cells was determined by AB/PAS staining. Histological injury was evaluated as a combined score of inflammatory cell infiltration (score 0–3) and mucosal damage (score 0–3), as previously described (Ren et al., 2020).

Healthy male BALB/c mice (6-week-old, 20–22 g) were used to establish tumor-bearing animal model. Briefly, CT26 colon cancer cells (1 × 10^6^ in 100 μL of PBS) were harvested and subcutaneously injected into the right flank of each mouse. Three days later, all tumor-bearing animals were divided into the following groups (*n* = 6 mice per group), control group, CPT11 group, and CPT11 + berberine group. At the end of experiment, all of the mice were euthanized under anesthesia and the tumors were removed and weighed. Tumor volume was then determined by measuring length (L, mm) and width (W, mm) and applying the following formula: V = 0.5 × L × W^2^.

### Intestinal Permeability Analysis

To investigate intestinal permeability *in vivo*, we used FITC-dextran (MW: 3,000–5,000, Shanghai Aladdin Biochemical Technology Co., Ltd, China) as a tracer. In brief, after fasting for 12 h, mice were administered with FITC-dextran (44 mg/100 g body weight). 4 h later, blood was collected upon euthanasia and allowed to clot for 1 h. After centrifugation (3,000 rpm, 15 min), serum was collected and diluted with the same volume of PBS. Finally, the FITC-dextran concentration was determined by spectrophotofluorometry (Fluoroskan Microplate Fluorometer, Thermo Fisher Scientific) at an excitation wavelength of 480 nm and an emission wavelength of 520 nm. We also determined the serum levels of lipopolysaccharide (LPS) and diamine oxidase (DAO) with ELISA kits (Yingxin laboratory Co., Ltd, Shanghai, China) in accordance with the manufacturer’s instructions (n = 5–6 per group).

### Measurement of Trans-Epithelial Electric Resistance and Permeability *in Vitro*

NCM460 cells (1.5 × 10^5^ cells/ml) were seeded into transwell cell culture chambers (6.5 mm diameter inserts, 3.0 μm pore size) (Corning Costar, Cambridge, MA) and the growth medium changed every other day. A NCM460 cell monolayer was formed after 10 days of culture. Then, NCM460 cells were administrated with berberine (50 μM), SN38 (250 nM), and SN38 (250 nM) plus berberine (50 μM), for 24 h. The TEER values were then determined with an ohmmeter and chopstick electrodes (Millipore ESR-2; Burlington, MA, United States). Data are presented as the resistance per unit area and was calculated by dividing resistance values by the effective membrane area. Inserts without cells were used as blanks.

The permeability of the NCM460 cell monolayer was determined by analyzing the flux in FITC-dextran. FITC-dextran (1 mg/ml) was added to the apical compartment of the insets. After 6 h of incubation, we collected aliquots of basolateral medium to allow us to measure fluorescence at 480 nm excitation and 520 nm emission wavelengths.

### Immunofluorescence Staining

Caco-2 cells were seeded onto coverslips in a 24-well plate at a density of 4 × 10^5^ cells/well and grown to full confluence. After treatment with berberine (50 μM), SN38 (250 nM), and SN38 (250 nM) plus berberine (50 μM), for 24 h, cells were washed three times with PBS and fixed with 4% paraformaldehyde for 10 min at room temperature. Then, cells were permeabilized with 0.1% Triton X-100 for 5 min and then blocked with 3% BSA for 1 h at room temperature. Caco-2 cells were then incubated overnight with rabbit anti-Occludin antibody (1:50, #A2601, ABclonal) at 4°C in the dark. Then, coverslips were washed and incubated with Alexa Fluor 594 goat anti-rabbit IgG secondary antibody (1:200, #AS039, ABclonal) for 60 min at room temperature in the dark. Finally, cells were counterstained with 4′,6-diamidino-2- phenylindole (DAPI) (Beyotime, Jiangsu, China) for 5 min at room temperature and visualized with a fluorescence microscope (Olympus CKX41, Tokyo, Japan).

### Immunoblotting Analysis

Proteins were extracted from colon tissues (1–1.5 cm proximal to the anus) and cultured NCM460 cells by homogenization in lysis buffer containing protease and phosphatase inhibitor cocktail tablets (Roche Diagnostics GmbH, Mannheim, GER). The supernatant was collected after centrifugation (4°C, 12,000 g, 15 min). Proteins (30 μg) were separated by 10% SDS-PAGE and transferred onto a PVDF membrane. The membrane was blocked in 5% (w/v) skimmed milk for 2 h at room temperature and then immunoblotted with primary antibody. Then, blots were washed and incubated with a HRP-coupled secondary antibody at room temperature. Finally, blots were visualized by enhanced chemiluminescence (ECL) detection reagents. Protein expression levels were then analyzed by a GS-700 imaging densitometer (Bio-Rad, CA). β-actin was used as an internal control.

### Quantitative Real-Time PCR Analysis

Total RNA was extracted using TRIzol Reagent (Life Technologies) according to the manufacturer’s protocol. Complementary DNA (Cdna) was reverse transcribed using the SuperScript Ⅱ Reverse Transcriptase kit (Thermo Fisher Scientific). Real-time PCR was performed using SYBR Green qPCR Master Mix (Applied Biosystems). Primers for the inflammatory mediators and internal reference were as follows: iNOS: forward, 5′-GTC​CTA​CAC​CAC​ACC​AAA​CT-3′, reverse, 5′-ATC​TCT​GCC​TAT​CCG​TCT​C-3′; IL-8: forward, 5′-ATT​CAT​TCC​TCT​CAA​ACT​CAT​T-3′ reverse, 5′-GCC​AAC​AGT​AGC​CTT​CAC-3’; Interleukin 1 beta (IL-1β): 5′-ATT​GTG​GCT​GTG​GAG​AAG​AAG​A-3′, reverse, 5′-TGA​AGG​AAA​AGA​AGG​TG-3′; TNF-α: forward, 5′-CTC​TTC​TCA​TTC​CTG​CTT​GT-3′, reverse, 5′-GTG​GTT​TGT​GAG​TGT​GAG​G-3′; Occludin: forward, 5′-ATG​TCC​GGC​CGA​TGC​TCT​C-3′, reverse, 5′-TTT​GCT​GCT​CTT​GGG​TCT​GTA​T-3’; ZO-1: forward, 5′-GCC​GCT​AAG​AGC​ACA​GCA​A-3′, reverse, 5′-TCC​CCA​CTC​TGA​AAA​TGA​GGA-3’; Claudin-7: forward, 5′-GGC​CAC​TCG​AGC​CTT​AAT​GGT​G-3′ reverse, 5′- CCT​GCC​CAG​CCG​ATA​AAG​ATG​G-3’; β-actin: forward, 5′-GGG​AAA​TCG​TGC​GTG​AC-3′, reverse, 5′-AGG​CTG​GAA​AAG​AGC​CT-3′. qPCR was then performed with the Takara SYBR Green Master Mix Kit and was quantitatively analyzed using the ABI Prism 7900HT Sequence Detection System (Life Technologies, Carlsbad, CA, United States) in accordance with the manufacturer’s instructions. We used β-actin as an internal control.

### GUS Enzyme Activity Assay and Inhibitory Kinetic Analysis *in Vitro*

The *in vitro* inhibition of bacterial GUS enzymes was assessed by a fluorescent GUS substrate 4-methylumbelliferyl-β-D-glucuronide (4-MUG) (CAS: 6160-80-1, MW: 352.29, ShangHai YuanYe Bio-technology Co., Ltd, China). Reactions consisted of 40 μL of GUS (500 ng/ml), 30 μL of 4-MUG (500 μM), various concentrations of berberine (0, 1.25, 2.5, 5, 10, 20, 40, 60, 80, 100, 200 μM), and assay buffer [75 mM, phosphate buffer, (pH 6.8)]. Reactions were initiated by the addition of 4-MUG and were then incubated for 30 min at 37°C. Then, the end point absorbance was determined by a microplate reader with an excitation wavelength of 350 nm and an emission wavelength of 450 nm.

Next, the inhibitory activity of berberine against *E. coli* β-glucuronidase was carefully investigated. Inhibition constant (Ki) values were determined by applying various concentrations of 4-MUG in the presence or absence of different concentrations of berberine. To determine the inhibition kinetic type (competitive inhibition, non-competitive inhibition, or uncompetitive), multiple concentrations of 4-MUG and various berberine concentrations were utilized to determine the corresponding reaction rates. The inhibition kinetic type was determined by identifying the intersection point in Dixon and Lineweaver-Burk plots. A second plot, featuring slopes from the Lineweaver-Burk plot *versus* inhibitor concentrations, was then used to calculate the corresponding inhibition parameter (Ki) value.

### GUS Enzyme Activity Assay and GUS-Producing Bacteria Detection in Faeces

The GUS activity in fecal was monitored by the absorbance at 405 nm from the p-nitrophenol release using 4-Nitrophenyl β-D-glucopyranoside (PNPG) at the substrate. Briefly, frozen fecal pellets were homogenized in potassium phosphate (PB) buffer (pH 7.4, 0.01 M). Then the supernatant of all samples were collected after centrifugation (10,000×g for 20 min) and total protein concentration was determined using the pierce BCA Protein Assay Kit (Yeasen Biotech Co., Ltd, Shanghai, China). Subsequently, fecal homogenate from all groups containing 100 μg/ml protein (20 μL) was incubated with 0.5 mM PNPG (20 μL) and PB buffer (80 μL) at 37°C for 30 min. After incubation, 250 μL sodium hydroxide (NaOH) (0.5 M) was added to stop the reaction, and the absorbance was measured at 405 nm by a microplate reader.

GUS-producing bacteria was detected by 4-Methylumbellifery-β-D-Glucuronide (4-MUG) agar culture plate. In brief, the weighed fecal pellets (50-70 mg) were homogenized and centrifuged at 2000 rpm for 5 min. Then, centrifugation of the isolated supernatant was centrifuged at 10,000 rpm for 20 min, and the precipitation was collected then used for the GUS-producing bacteria detection. Finally, the precipitation of all samples were suspended in physiological saline (500 μL), then coated on the culture plate of nutrient agar plus 4-methylumbellifery β-D-glucuronide (4-MUG) and incubated for 12 h at 37°C. GUS-producing bacteria was detected by UV-light in 366 nm.

### Imaging of Intestinal GUS Activity

Berberine (50 mg/kg), or an equivalent volume of vehicle control (200 μL of 0.5% MC-Na) was administered into mice (*n* = 3/group) orally for three consecutive days. After 30 min of the final administration of berberine, mice were administered (by oral gavage) with fluorescein Di-β-D-Glucuronide (FDGlcU, 7.3 μmol/kg, 0.1 ml per mouse) (CAS: 129,787-66-2, BioRuler, CT, United States). Mice were euthanized immediately prior to imaging with a IVIS Spectrum image system (Bruker) (470 nm excitation/535 nm emission filters). GUS activity was quantified by measuring the mean fluorescence in a given region of interest after subtracting the background; this was held constant throughout the imaging period.

### Molecular Docking Simulation

Molecular docking experiments were carried out with AutoDock 4.2.6 program to investigate the interaction between berberine and GUS protein. The three-dimensional structure of GUS protein (PDB code: 3K46 (Wallace et al., 2010). resolution: 2.0 Å) was obtained from the Protein Data Bank. The initial structure was then prepared using AutoDockTools 1.5.6, preserving the original charges of the protein and generating a pdbqt file for docking (Sanner, 1999). The two-dimensional structure of berberine was downloaded from the PubChem database. The MOPAC program was then used to optimize the structure and calculate the PM3 atomic charge (Stewart, 1990; Stewart, 2004). The structure of berberine was also prepared by AutoDock Tools 1.5.6, and the corresponding pdbqt file was generated for docking. The hydrophobic region of GUS was chosen as the binding pocket for docking. The coordinates of the grid box center were then determined (−14.604, −32.129, 47.104) and the number of grid points in the XYZ of grid box was set to 80 × 60 × 60. The grid spacing was 0.375 Å and the number of GA runs was set to 100. The remaining parameters were set to default.

### Statistics

Data were compared between multiple groups by one-way analysis of variance (ANOVA) and GraphPad Prism 7 software (GraphPad Software, La Jolla, CA, United States). Data are shown as mean ± SD. p-values < 0.05 (two-sided) were considered significant (*p < 0.05, **p < 0.01, ***p < 0.001).

## Results

### Berberine Prevented CPT11-Induced Intestinal Mucositis

**FIGURE 1:**
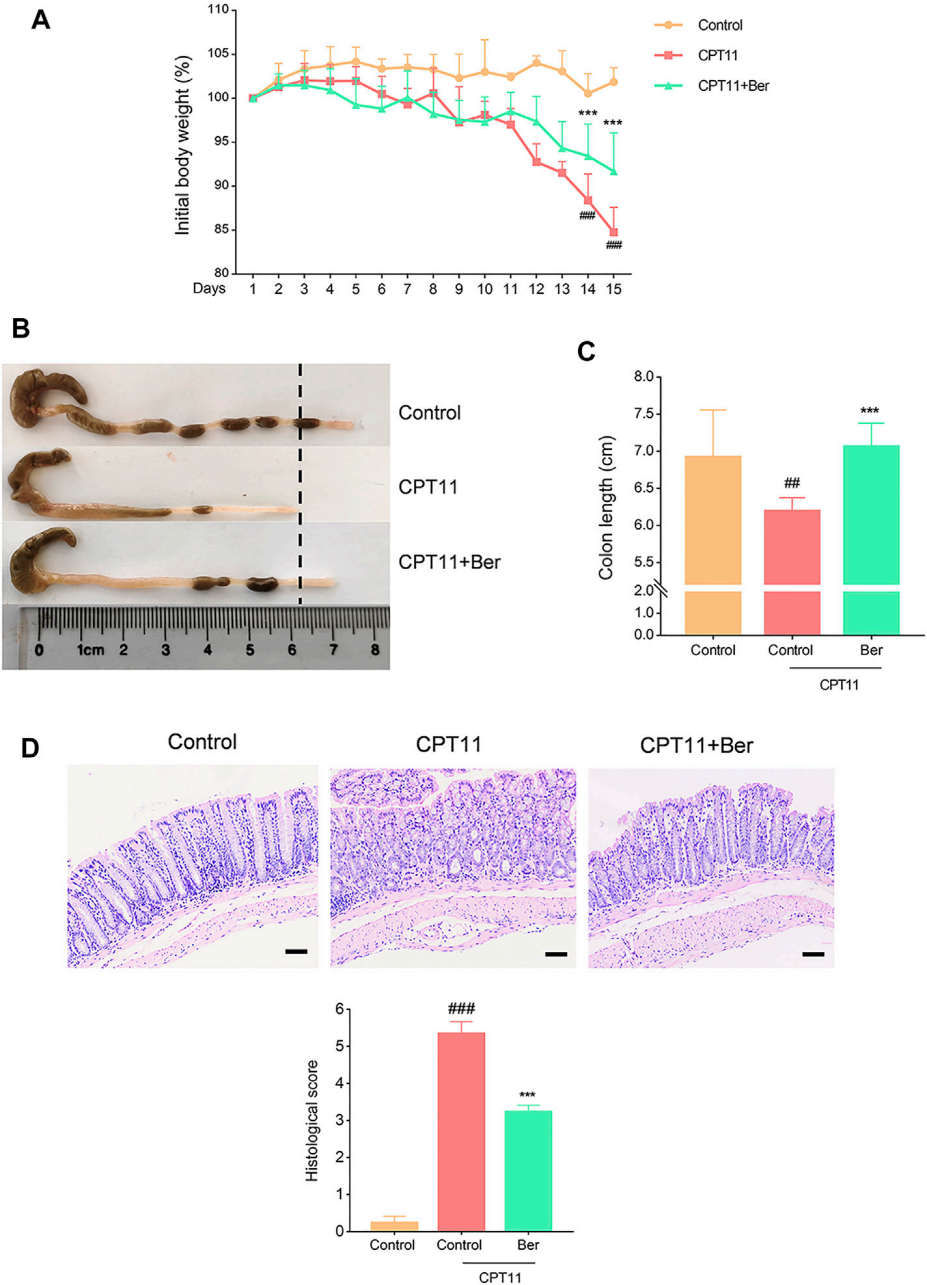
Berberine ameliorated body weight loss, colon shortening, and histopathologic injury, in mice with CPT11-induced intestinal mucositis. **(A)** Changes in body weight in mice with CPT11-induced mucositis. The data are plotted as a percentage of the original body weight. Macroscopic observations **(B)** and the assessment of colon shortening **(C)** after berberine treatment. **(D)** Representative H&E-stained colon sections and histological scores. Scale bars correspond to 50 μm. Data are expressed as the mean ± SD (*n* = 6 per group). ##*p* < 0.01 *vs.* the control group; ***p < 0.001 *vs*. CPT11-treated group.

Compared with the vehicle treatment control mice, the mice receiving CPT11 treatment exhibited significant body weight loss (Figure 1A). Berberine treatment attenuated the CPT11-induced body weight loss. Furthermore, colon shortening was identified as an indirect symptom, thus indicating the intensity of mucosal inflammation (Ren et al., 2020). As anticipated, the CPT11-induced colon shortening was remarkable, but was significantly mitigated by berberine treatment (Figures 1B,C). Further histopathological analysis showed that CPT11 caused severe intestinal tissue damage, including severe epithelial injury, loss of architecture, muscle thickening, and neutrophil infiltration. Berberine treatment mitigated the CPT11-induced loss of mucosal architecture, ulceration, and cellular infiltration (Figure 1D).

### Berberine Inhibited the Production of Inflammatory Mediators and Promoted the Expression of Tight Junction Proteins in Mice With Mucositis

**FIGURE 2:**
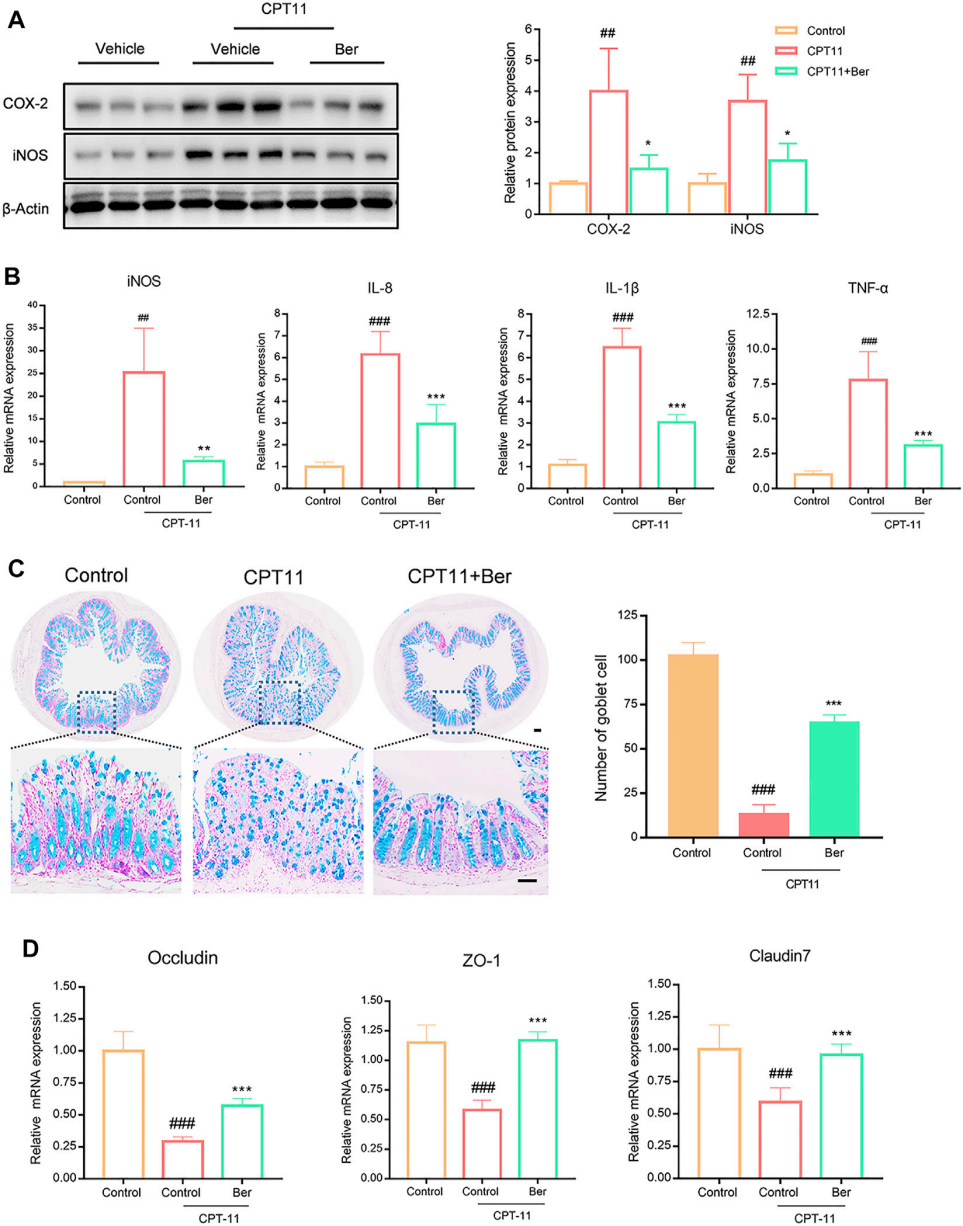
Berberine inhibited the expression levels of pro-inflammatory mediators and promoted the mRNA expression levels of tight junction protein *in vivo*. **(A)** The effect of berberine on the expression of pro-inflammatory mediators (COX-2 and iNOS) in colonic tissues. Representative western blots and quantitative analysis of COX-2 and iNOS proteins. **(B)** mRNA expression levels of iNOS, IL-8, IL-1β, and TNF-α in colon tissue, as determined by qRT-PCR. **(C)** Representative Alcian blue (AB)/(Periodic Acid-Schiff stain) PAS-stained colonic tissue sections (Scale bars, 100 μm) and goblet cell number. **(D)** mRNA expression levels of Occludin, ZO-1, and Claudin-7, in colon tissue, as determined by qRT-PCR. All mRNA expression levels were normalized to β-Actin. Data are expressed as the mean ± SD (*n* = 3 per group). ##*p* < 0.01, ###*p* < 0.001 *vs.* the control group; *p < 0.05, **p < 0.01, ***p < 0.001 *vs.* CPT11 + berberine treated group.

**FIGURE 3:**
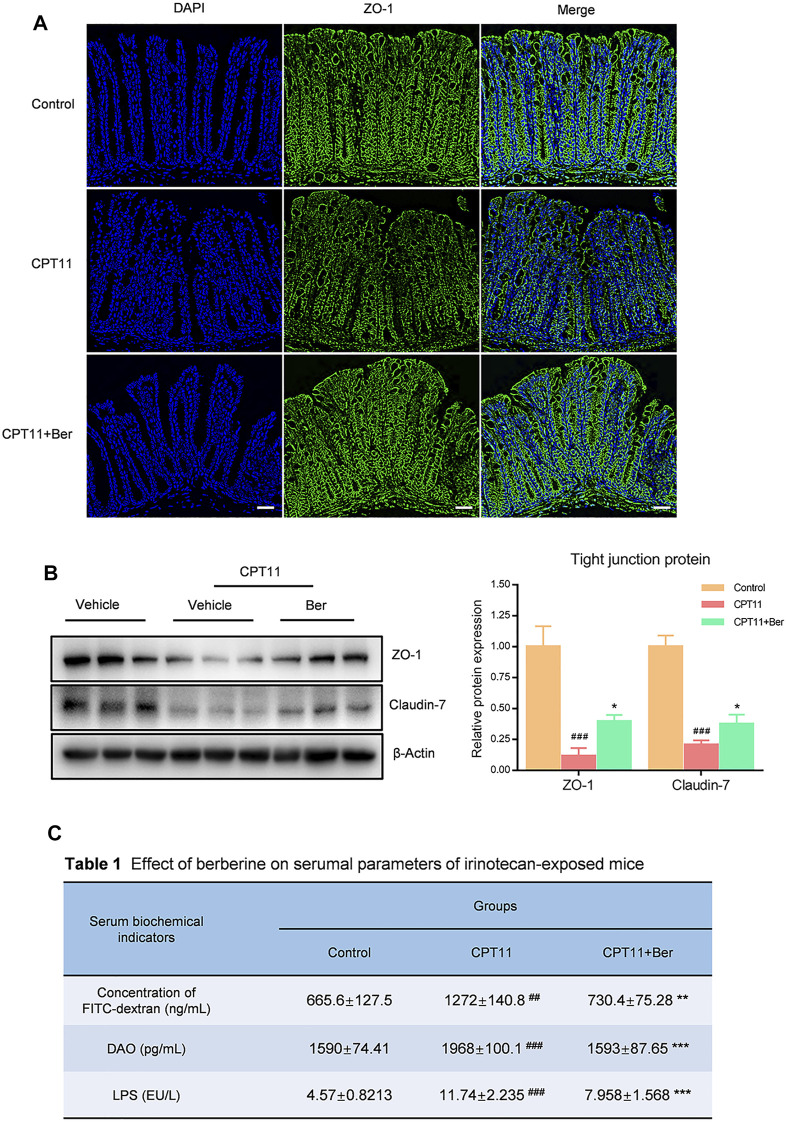
Berberine protected the integrity of the intestinal barrier in mice with mucositis. **(A)** Immunofluorescence staining of the colonic tissues for ZO-1 protein (green). Nuclei were stained with DAPI (blue). **(B)** The effect of berberine on the expression levels of tight junction proteins (ZO-1 and Claudin-7) in colonic tissues. Representative western blots and quantitative analysis of the proteins. **(C)** The fluorescence intensity of FITC-dextran was detected by fluorescence spectroscopy (*n* = 4–5). The serum levels of LPS and DAO were determined by ELISA. Data are expressed as the mean ± SD (*n* = 3 per group). ##*p* < 0.01, ###*p* < 0.001 *vs.* the control group; *p < 0.05, **p < 0.01, ***p < 0.001 *vs.* CPT11 + berberine treated group.

We next investigated the expression levels of multiple inflammatory mediators. The treatment of mice with CPT11 for 14 days significantly increased the levels of COX-2, iNOS, IL-8, IL-1β, and TNF-α in colon tissues (Figures 2A,B). However, the upregulated levels of these inflammatory mediators, as induced by CPT11, were significantly reduced by berberine treatment. Additionally, we observed a significant decrease in the number of goblet cells in the colons of mice receiving CPT11 (Figure 2C). Berberine significantly increased the number of goblet cells in CPT11-treated mice. Furthermore, berberine treatment increased the levels of the tight junction proteins ZO-1 and Claudin-7 (Figure 3B), as well as the mRNA levels of Occludin, ZO-1, and Claudin-7 in CPT11-induced intestinal mucositis mice (Figures 2B,C).

### Berberine Protected the Integrity of the Intestinal Barrier in Mice With Mucositis

To determine the effects of berberine on the mucosal barrier, we performed immunofluorescence staining to examine the expression of ZO-1 in the colon of CPT11-induced mucositis mouse. CPT11-treated mice showed a disruption of the intestinal intercellular structure and a reduced expression of ZO-1 in the colon (Figure 3A). In addition, intestinal permeability in mouse plasma was assessed by FITC-dextran fluorescence (Table). Mice exposed to CPT11 exhibited higher FITC positive signals, indicating that the intestinal barrier had been disrupted by CPT11. Berberine treatment reduced the FITC signal, thus indicating an improvement in the barrier integrity of CPT11-treated mice. Similar results were observed for the concentrations of lipopolysaccharide (LPS) and diamine oxidase (DAO) (Table 1), both of which are serum indicators of mucosal injury. Collectively, these data indicate that berberine effectively prevents CPT11-induced dysfunction in the intestinal barrier.

### Berberine Protected the Integrity of the Intestinal Barrier in Epithelial Cells

**FIGURE 4:**
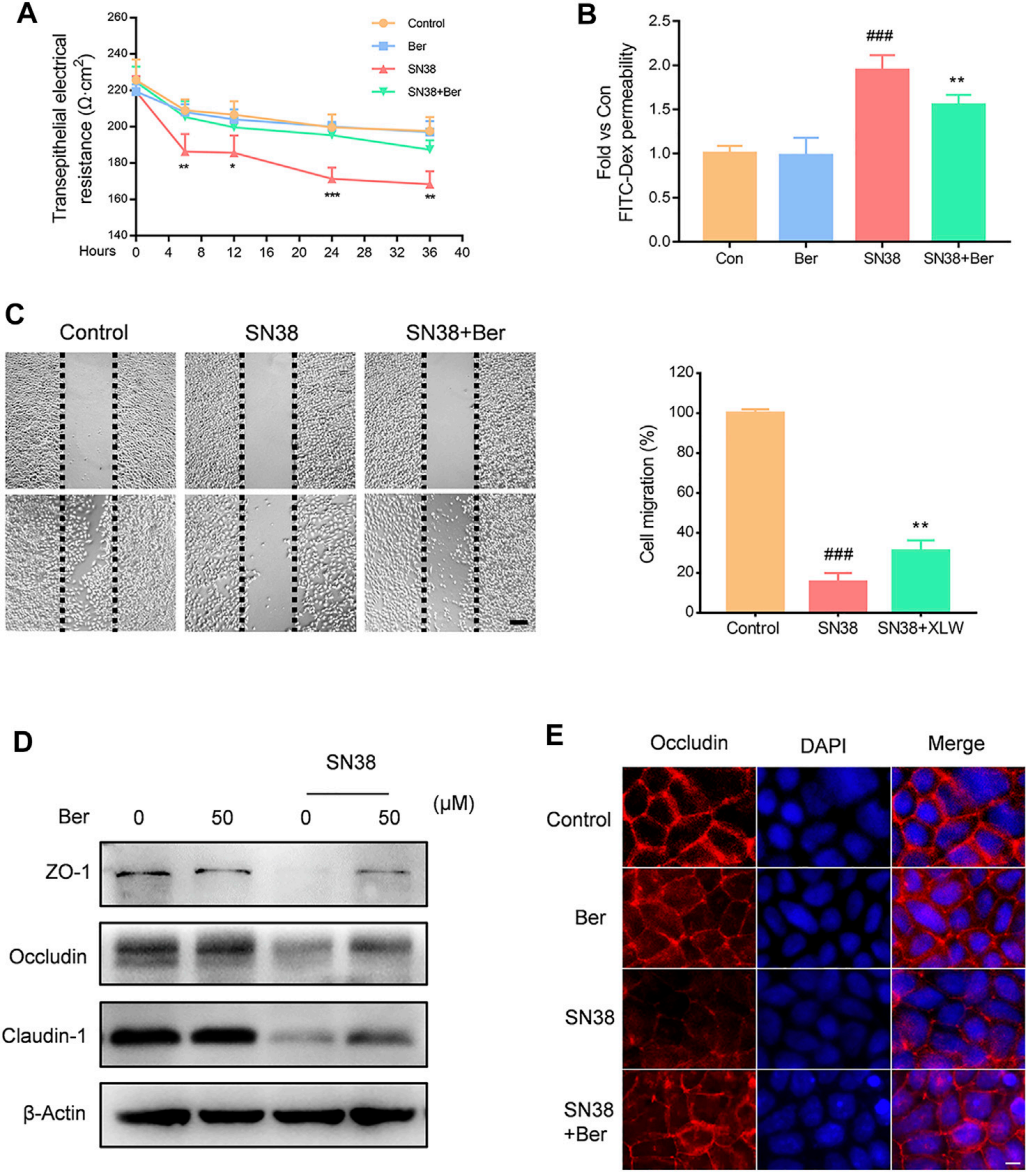
Effect of berberine on SN38-induced disruption of barrier function *in vitro*. **(A)** NCM460 cell monolayers were treated with berberine (50 μM), SN38 (250 nM) or SN38 (250 nM)) + Ber (50 μM) for 4, 12, 24, and 36 h, respectively; then, we measured trans-endothelial electrical resistance (TEER). **(B)** The flux of 4 kDa FITC-dextran was measured by fluorescence spectroscopy. **(C)** Representative images of scratch wound assays for NCM460 cell monolayers at 0 and 24 h after scratching. **(D)** The effect of berberine on the expression levels of tight junction proteins (ZO-1, Occludin, and Claudin-7) in NCM460 cells. **(E)** The expression of Occludin (red) was evaluated by immunofluorescence. Nuclei were stained with DAPI (blue). Data were expressed as the mean ± SD (*n* = 3 per group). ##*p* < 0.01, ###*p* < 0.001 *vs.* the control group; *p < 0.05, **p < 0.01, ***p < 0.001 *vs.* CPT11 + berberine treated group.

We further evaluated the effects of berberine on the intestinal barrier *in vitro*. NCM460 human intestinal epithelial cells were incubated with berberine, SN38, and SN38 + berberine, respectively. We found that berberine significantly suppressed the SN38-induced trans-endothelial electrical resistance (TEER) decline (Figure 4A), as well as the increase in FITC-Dex permeability (Figure 4B). Furthermore, SN38 effectively inhibited the wound healing of NCM460 cells; however, berberine treatment improved this situation (Figure 4C). Then, we assessed the expression levels of tight junction proteins. We found that treatment of NCM460 cells with berberine significantly improved the expression levels of ZO-1, Occludin, and Claudin-1 (Figure 4D). Then, Caco-2 cells were cultured to form a monolayer to simulate the intestinal barrier *in vitro*. Immunofluorescence assay showed that the level of Occludin protein was significantly decreased in Caco-2 monolayer treated with SN38. However, pre-incubation with berberine increased the Occludin expression level in Caco-2 cells (Figure 4E). Together, these results indicate that berberine protects the barrier function from SN38-induced injury *in vitro*.

### Berberine Reduced Intestinal GUS Activity and the Production of SN38

**FIGURE 5:**
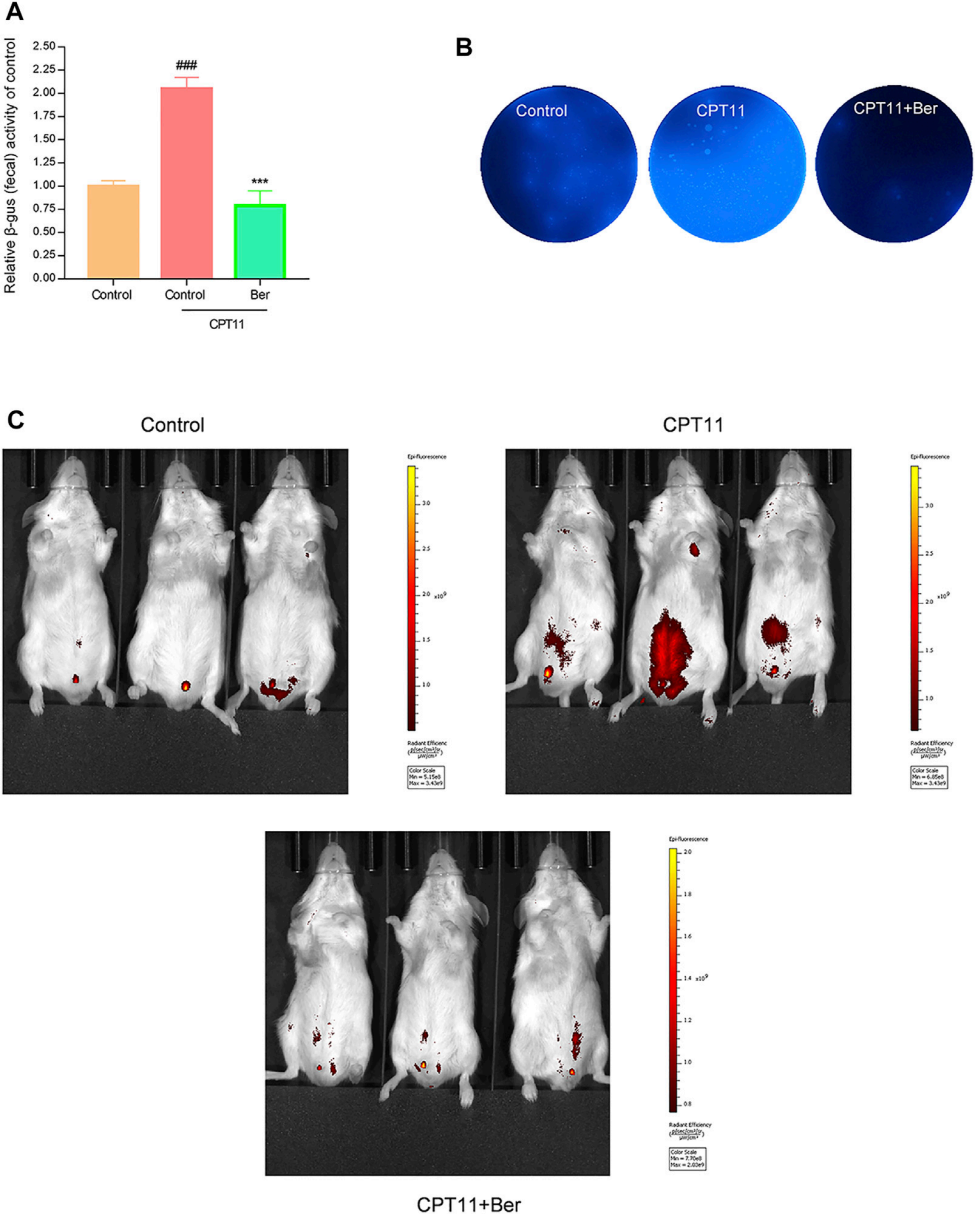
Berberine inhibited intestinal GUS activity. **(A)** Fecal pellets were collected to evaluate the GUS activity in mice after CPT11 and CPT11 + berberine treatment by 4-MUG assay. **(B)** The assessment of fecal pellets for GUS-producing bacteria was determined by culture with 4-MUG agar medium. **(C)** Mice were treated with CPT11 or CPT11 + berberine for 14 days; GUS activity was then imaged using the fluorescent substrate FDGlcU. Data were expressed as the mean ± SD (*n* = 3 per group). ##*p* < 0.01, ###*p* < 0.001 *vs.* the control group; **p < 0.01, ***p < 0.001 *vs.* CPT1 + berberine treated group.

Intestinal bacterial β-glucuronidase GUS catalyzes SN38G to SN38, and SN38 is considered to be the cause of CPT11-induced intestinal mucositis. To explore whether administration of berberine affacts the activity of GUS in fecal samples, we performed the 4-Nitrophenyl β-D-glucopyranoside (PNPG) assay using fecal lysates. The total GUS activity increased significantly after 14 days of CPT11 exposure (Figure 5A). However, berberine treatment significantly reduced the GUS enzyme activity in mice that had been exposed to CPT11 (*p* < 0.001) (Figure 5A). Subsequently, we used 4-Methylumbellifery-β-D-Glucuronide (4-MUG) agar to analyze GUS-producing bacteria. The results showed that berberine markedly reduced the levels of GUS-producing bacteria in fecal samples (Figure 5B). Furthermore, a non-fluorescent GUS probe (FDGlcU) was used to verify the attenuated effects of berberine on GUS activity in the intestine. As shown in Figure 5C, mice receiving FDGlcU (7.3 μmol/kg) by oral gavage and exposed to CPT11 showed increased fluorescent signal, which represents increased intestinal GUS activity. However, the fluorescent signal in CPT11 treatment mice was slighter following berberine administration. On the other hand, the LC-MS technology was used to determine the concentration of CPT11 and SN38 in feces. Relatively low levels of SN38 in faecal samples were detected in berberine + CPT11-treated mice compared with CPT11-treated mice (Supplementary Figure S1). Collectively, these data indicate that berberine effectively inhibits bacterial GUS activity, reduces GUS-producing bacteria, and decreases the production of SN38 in a mouse model of CPT11-induced intestinal mucositis.

### Confirmation of the Inhibitory Properties of Berberine on Bacterial GUS Enzyme by Molecular Docking Analysis

**FIGURE 6:**
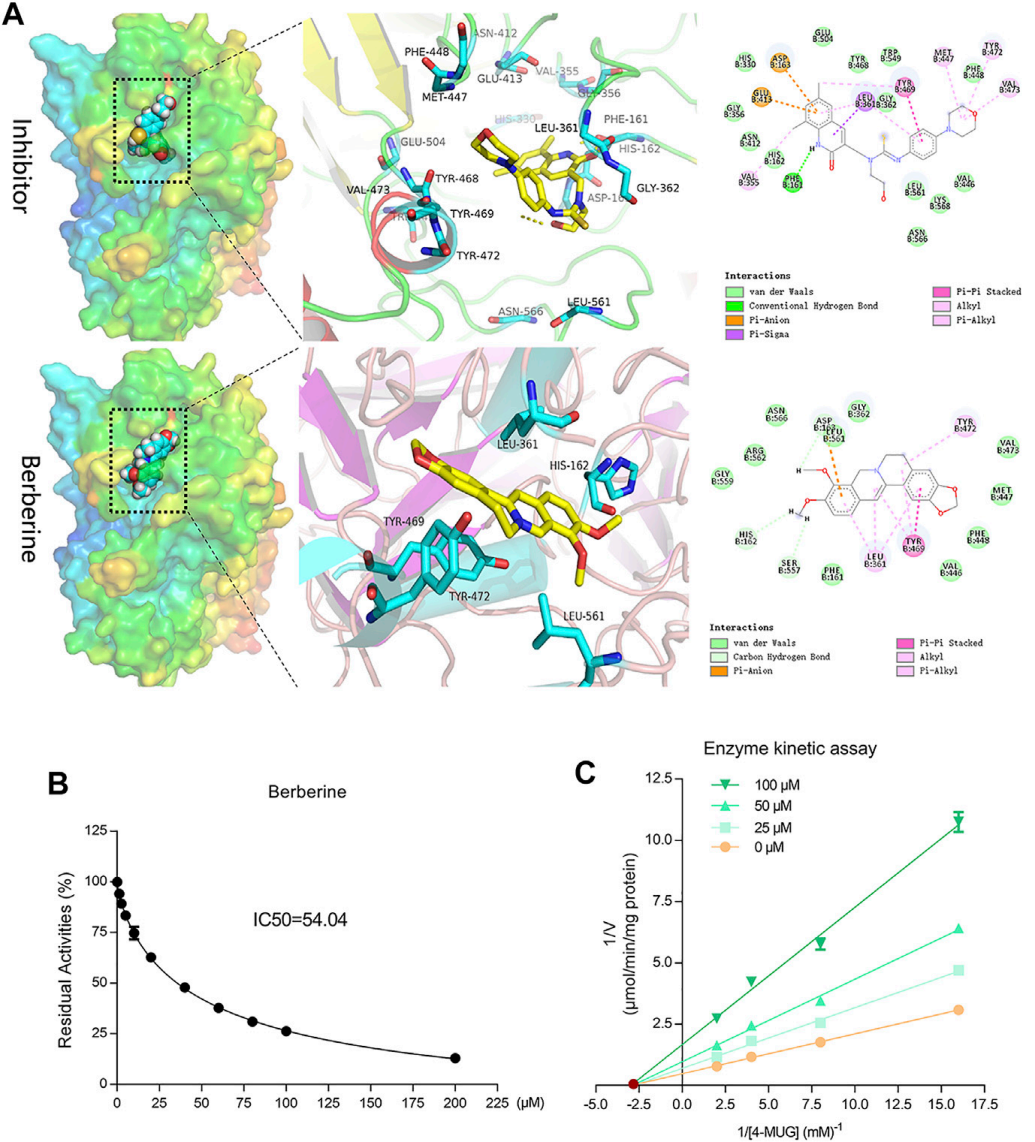
Berberine inhibited GUS enzyme in a non-competitive manner. **(A)** Active site interactions for berberine or inhibitor-bound *E. coli* GUS. **(B)** Berberine inhibited GUS activities in a dose-dependent manner. **(C)** Lineweaver-Burk plots for berberine against GUS.

Next, we performed molecular docking assays to evaluate the potential binding of berberine to *E. coli* GUS enzyme. Inhibitor 1, as a positive control compound, is an inhibitor of GUS enzyme that has been used in previous studies (LoGuidice et al., 2012; Saitta et al., 2014; Bhatt et al., 2020). We found that both berberine and inhibitor 1 played roles in the catalytic cavity of GUS with relatively low binding energies of −7.8 and −8.6 kcal/mol, respectively. Berberine formed three hydrogen bonds between the hydroxyl groups at C-9 and C-10, and several amino acid residues (Asp163, His162, and Ser 557) (Figure 6A). In addition, berberine associated with Leu561, Leu361, Tyr469, and Tyr472 via Pi-Anion, Alkyl, Pi-Alkyl, and Pi-Pi stacked interactions; these interactions may contribute to the binding affinity between *E. coli* GUS and berberine. Subsequently, a 4-MUG assay demonstrated the inhibitory effects of berberine on GUS enzyme with an IC_50_ value of 54.04 ± 5.104 μM (Figure 6B). To further explore the inhibitory mechanisms of berberine on GUS, we prepared Lineweaver-Burk plots (substrate concentration ^−1^ *versus* velocity ^−1^) and found that berberine acted in a non-competitive manner against GUS-mediated 4-MUG hydrolysis (Figure 6C). These results indicate that berberine functions as a potential non-competitive inhibitor against bacterial GUS enzyme.

### Berberine Maintained the Anti-Tumor Effects of CPT11 in Tumor-Bearing Mice

**FIGURE 7:**
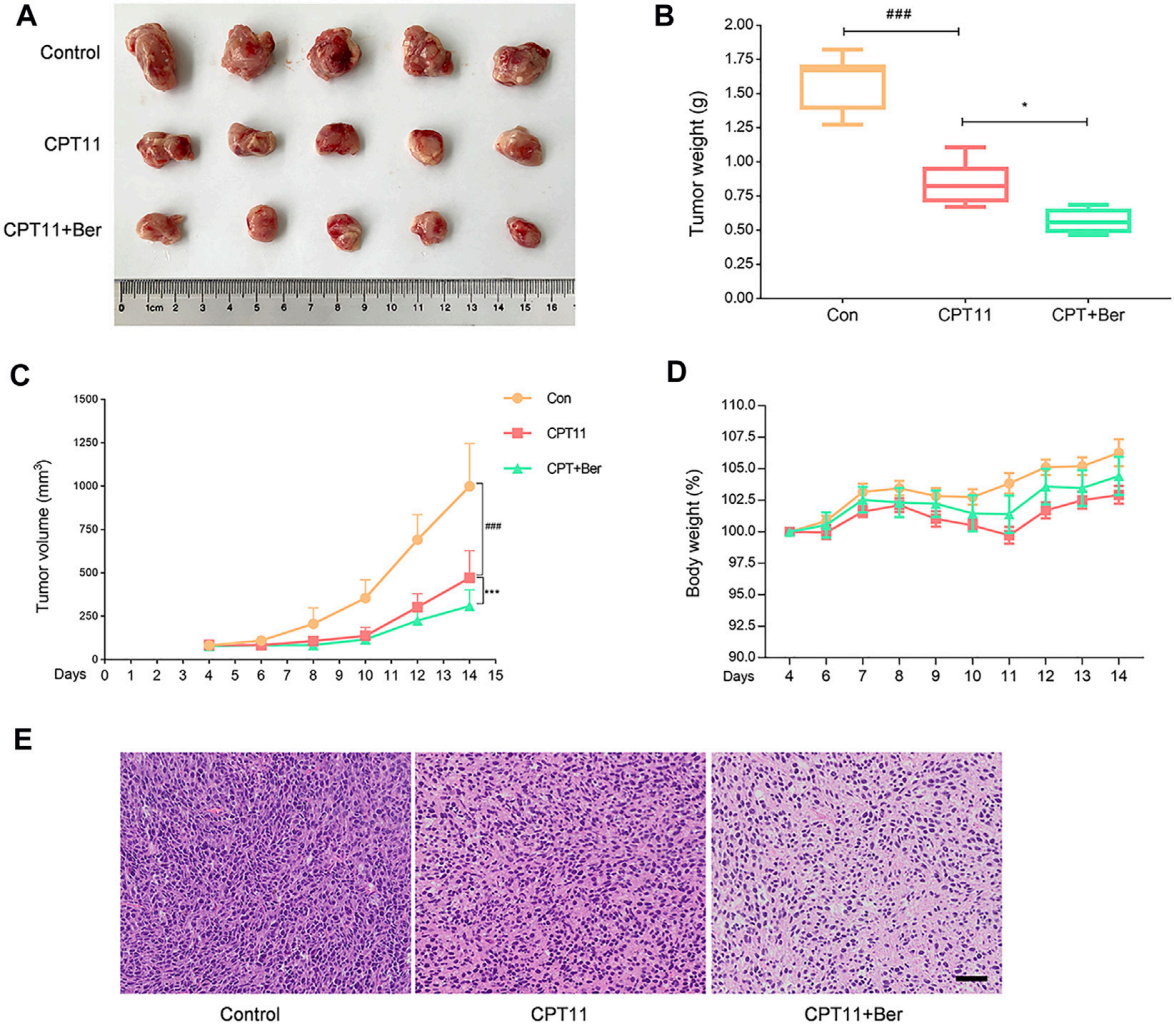
Berberine (50 mg/kg) improved the anti-tumor effects of CPT11 in a colon cancer xenograft model. **(A)** Image of colorectal tumor in each group. **(B)** Tumor weights were measured after animals were euthanized. **(C)** Tumor volumes were measured during the test period. **(D)** Changes in body weight were monitored throughout the study. **(E)** Images of hematoxylin and eosin staining in tumor tissues. Data are expressed as the mean ± SD (*n* = 5 per group). ###*p* < 0.001 *vs.* the control group; ***p < 0.001 *vs.* CPT11 + berberine treated group.

Finally, we evaluated whether the inhibition of bacterial GUS by berberine affects the anti-tumor efficacy of CPT11 in tumor-bearing mice. A tumor xenograft mouse model was established by injecting mice with CT26 cells, a murine colorectal carcinoma cell line. As shown in Figure 7, compared with controls, the tumor weight (Figure 7B) and volume (Figure 7C) were significantly reduced after CPT11 treatment. Notably, the tumors in mice in the berberine + CPT11 co-treatment group grew more slowly when compared with those in the CPT11 alone treatment group. Then, we investigated the effect of berberine alone on tumor growth and found that berberine inhibited tumor growth in a colorectal cancer xenograft model (Supplementary Figure S2). Collectively, these data indicate that berberine inhibits intestinal GUS activity and attenuates CPT11-induced intestinal mucositis without impairing CPT11-mediated tumor regression.

## Discussion

In clinical practice, CPT11 is widely used for the treatment of metastatic or advanced solid tumors, such as gastric, pancreatic, ovarian, and colorectal cancers. However, CPT11-induced mucositis is common in cancer patients receiving chemotherapy. According to clinical data, up to 80% of cancer patients receiving CPT11 chemotherapy may undergo intestinal mucositis, involving diarrhea, bleeding, abdominal pain, vomiting, infections, malnutrition, and sepsis due to bacterial translocation (Bleiberg and Cvitkovic, 1996; Rubenstein et al., 2004). The failure of CPT11 chemotherapy is highly correlated with the occurrence of CPT11-mediated intestinal mucositis. Moreover, CPT11-induced mucositis is known to increase health care costs (mean medical cost for treating grade 3-4 CPT11-related diarrhea = $2000), hospital admission (32.3% of patients), and mortality rates (Dranitsaris et al., 2005). Up to now, anti-cholinergic agents, anti-diarrheal agents, and antibiotics, are the main options for preventing or treating CPT11-induced mucositis in the clinic, although these agents are associated with only limited treatment outcomes (Elad et al., 2020).

However, herbal medicines are being increasingly used to alleviate the adverse effects of cancer treatments or to enhance chemotherapeutic efficacy. A variety of herbs have been used as adjuvants to cope with the side effects of CPT11 chemotherapy, including PHY906, Ginseng, Curcumin, Gegen Qinlian (Wu et al., 2019; Wu et al., 2021), and Huanglian Jiedu (Chan et al., 2020). *Rhizoma coptidis* (Huanglian), a conventional herbal medicine with a history of use, consists mainly of berberine and other alkaloids (Wang et al., 2019). Berberine has received considerable levels of interest by virtue of its range of pharmacological activities, particularly in terms of pro-apoptotic and anti-inflammatory activities (Jin et al., 2016). In the present study, we demonstrated that berberine, as an adjuvant agent of CPT11 chemotherapy, reduced intestinal toxicity without abrogating its anti-colorectal cancer efficacy.

CPT11-induced intestinal mucositis involves a multifactorial and pathological course that includes intestinal epithelium damage, destruction of tight junctions, an increase in intestinal permeability, and ulceration of the bowel wall. In the present study, we used a widely used experimental model of CPT11-induced intestinal mucositis to investigate the intestinal toxicity of CPT11 chemotherapy (Cechinel-Zanchett et al., 2019). We observed a significant weight loss, colon shortening, and epithelial injury, in mice exposed to CPT11 treatment; however, administration of berberine improved these hallmarks of disease. Previous studies investigated the levels of inflammatory mediators during the pathogenesis of intestinal mucositis and found that several inflammatory cytokines were markedly increased in the colons of mice treated with CPT11 (Ribeiro et al., 2016). Correspondingly, in the present study, we observed increased levels of COX-2, iNOS, IL-8, IL-1β, and TNF-α in mice with CPT11-induced mucositis. The oral administration of berberine significantly reduced the production of these pro-inflammatory mediators. Furthermore, compelling evidence previously indicated that increased inflammatory cytokines levels are closely associated with the disruption of the intestinal mucosal barrier (Wardill et al., 2012; Suzuki, 2013). As key functional proteins in the mucosal barrier, tight junctions are multiple protein complexes that regulate intestinal permeability and control the movement of fluids, nutrients, microbes, and toxins, across the epithelium. Key findings from previous studies indicated that defective tight junctions cause mucosal barrier dysfunction and lead to the development of mucositis. Peripherally located zonular occludens (ZO) proteins are cytosolic scaffold proteins that interact with each other to anchor tight junction membrane proteins to the cytoskeleton (Noda et al., 2013). Claudins are the vital components of intercellular tight junctions and are responsible for paracellular solute flux (Will et al., 2008). As an essential transmembrane protein, Occludin is integral for tight junction integrity (Hanning et al., 2021). Our present studies identified a disruption of the intestinal intercellular structure and a reduced expression of ZO-1, Claudin-7, and Occludin in mice with mucositis. We also observed a reduced number of goblet cells in these mice. Furthermore, the serum concentrations of LPS and DAO, as well as the intestinal permeability, were all significantly increased in mice with mucositis. Subsequently, we observed that SN38 (the active metabolite of CPT11) caused significant injury to the barrier function of epithelial cells *in vitro*, including a decline in TEER, an increase in permeability, the retarded wound healing of NCM460 cells and a deficiency of tight junctions. Conversely, treatment with berberine efficiently maintained the levels and location of tight junctions in the intestinal epithelial layer both *in vitro* and *in vivo*, thus demonstrating that berberine treatment may have the potential to prevent against CPT11 or SN38-induced intestinal barrier dysfunction.

The structure of bacterial GUS has been known for decades; however, it was only recently that we began to understand its function in CPT11-induced intestinal toxicity. Bacterial GUS, a lysosomal exoglycosidase, can cleave SN38G (non-toxic) to SN38 (active) in the lumen of the bowel (Bailly, 2019). SN38G can be subject to fast renal clearance from the body. Bacterial GUS prolongs the clearance time of SN38 within the body and is, therefore, considered to be a direct promotor of CPT11-induced intestinal mucositis (Wallace et al., 2010). There has been significant interest over recent years in understanding how the inhibition of bacterial GUS can alleviate intestinal mucositis. Various inhibitors against *E. coli* GUS enzyme have been designed, which were shown to actively protect mice from CPT11-induced mucositis without causing cellular injury (Roberts et al., 2013; Pollet et al., 2017; Lin et al., 2021). Therefore, we performed molecular docking assays using *E. coli* GUS protein and found that berberine could enter a pocket domain of GUS with low binding energy. Subsequently, the 4-MUG assay confirmed the inhibitory activity of berberine (IC_50_ = 54.04 ± 5.104 μM) on GUS. By applying the PNPG assay, we found that berberine treatment also significantly reduced GUS enzyme activity in fecal samples when compared with a CPT11 alone treatment group. Furthermore, by using 4-MUG agar culture plates, a lower level of GUS-producing bacteria were detected in fecal samples of berberine + CPT11-treated group compared with CPT11 alone treatment group. In addition, FDGlcU-based *in vivo* imaging is widely used to detect the enzymic activity of intestinal bacterial GUS *in vivo* with a fluorescent signal generated from the enteric cavity that responds to GUS activity (Chen et al., 2017). In our present study, mice were administered with FDGlcU (7.3 μmol/kg) by oral gavage and then exposed to CPT11; these mice showed an up-regulation of GUS activity. However, berberine treatment significantly reduced the up-regulated fluorescence signal, thus indicating the effective inhibition of berberine against bacterial GUS activity in the intestines of mice. Further analysis of Lineweaver-Burk plots suggested that berberine might inhibit GUS enzyme by non-competitive inhibition. To confirm all the results above, we used LC-MS technology to determine the concentrations of CPT11 and SN38 in feces. As predicted, concentration of SN38 in the feces of berberine + CPT11-treated mice was lower than CPT11-treated mice. Collectively, berberine effectively inhibited bacterial GUS activity and reduced GUS-producing bacteria, thereby decreased the production of SN38 in a mouse model of CPT11-induced intestinal mucositis.

Furthermore, we established a tumor-bearing model using CT26 murine colorectal carcinoma cells to examine whether inhibition of bacterial GUS by berberine alters the anti-tumor efficacy of CPT11. Interestingly, our study showed that berberine enhanced CPT11-mediated tumor regression. This is supported by a recent study demonstrating that inhibition of intestinal GUS without impairing the anti-tumor efficacy of CPT11 (Cheng et al., 2019). Our further studies indicated that the synergetic effects of berberine on CPT11-mediated tumor regression could be associated with the anti-tumor effects of berberine itself. Notably, no obvious toxicity and side effects were observed in our *in vitro* and *in vivo* experiments, suggesting that berberine has the potential to be developed as an adjuvant, to compliment and/or improve the efficacy of CPT11 in cancer therapy.

## Conclusion

In the present study, we demonstrated that berberine significantly mitigated CPT11-induced intestinal mucositis. We revealed that the underlying mechanisms were related to the improved mucosal barrier function via inhibiting and reducing bacterial GUS enzyme. Specifically, berberine improved CPT11-induced intestinal mucositis without impairing the anti-tumor efficacy of CPT11, which could be associated with the anti-tumor effects of berberine itself and need further clarification. Our results provide new insights into the potential application of berberine for the prevention of chemotherapy-induced intestinal toxic side-effects.

## Data Availability

The original contributions presented in the study are included in the article/Supplementary Material, further inquiries can be directed to the corresponding authors.
