# Supplementary figures and images for: Berberine Improves Irinotecan-Induced Intestinal Mucositis Without Impairing the Anti-colorectal Cancer Efficacy of Irinotecan by Inhibiting Bacterial β-glucuronidase
Source: Front Pharmacol. 2021 Nov 2;12:774560. doi: 10.3389/fphar.2021.774560 (PMC8593678; doi:10.3389/fphar.2021.774560)

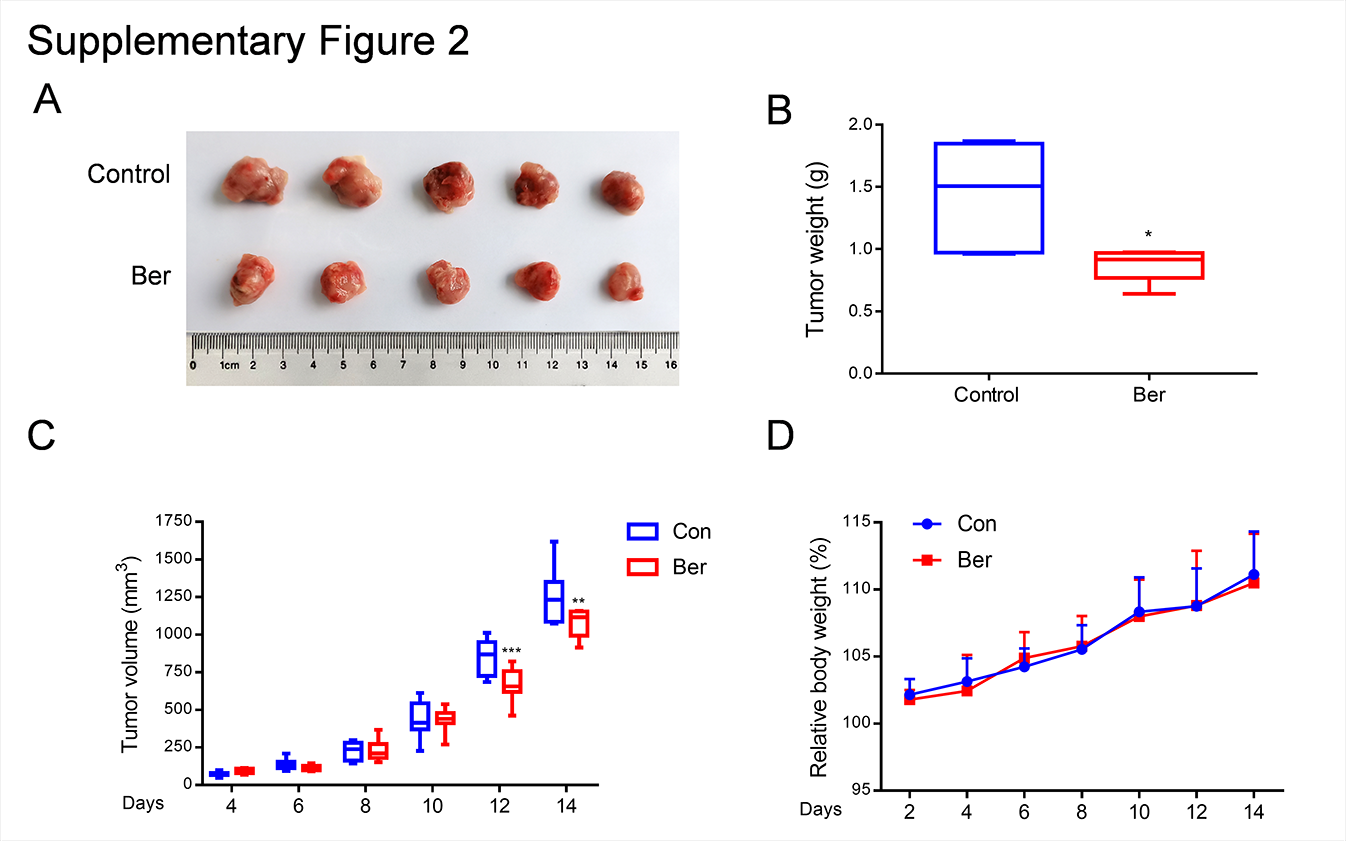

Supplement: Supplementary file 1 [file Image2.TIF]

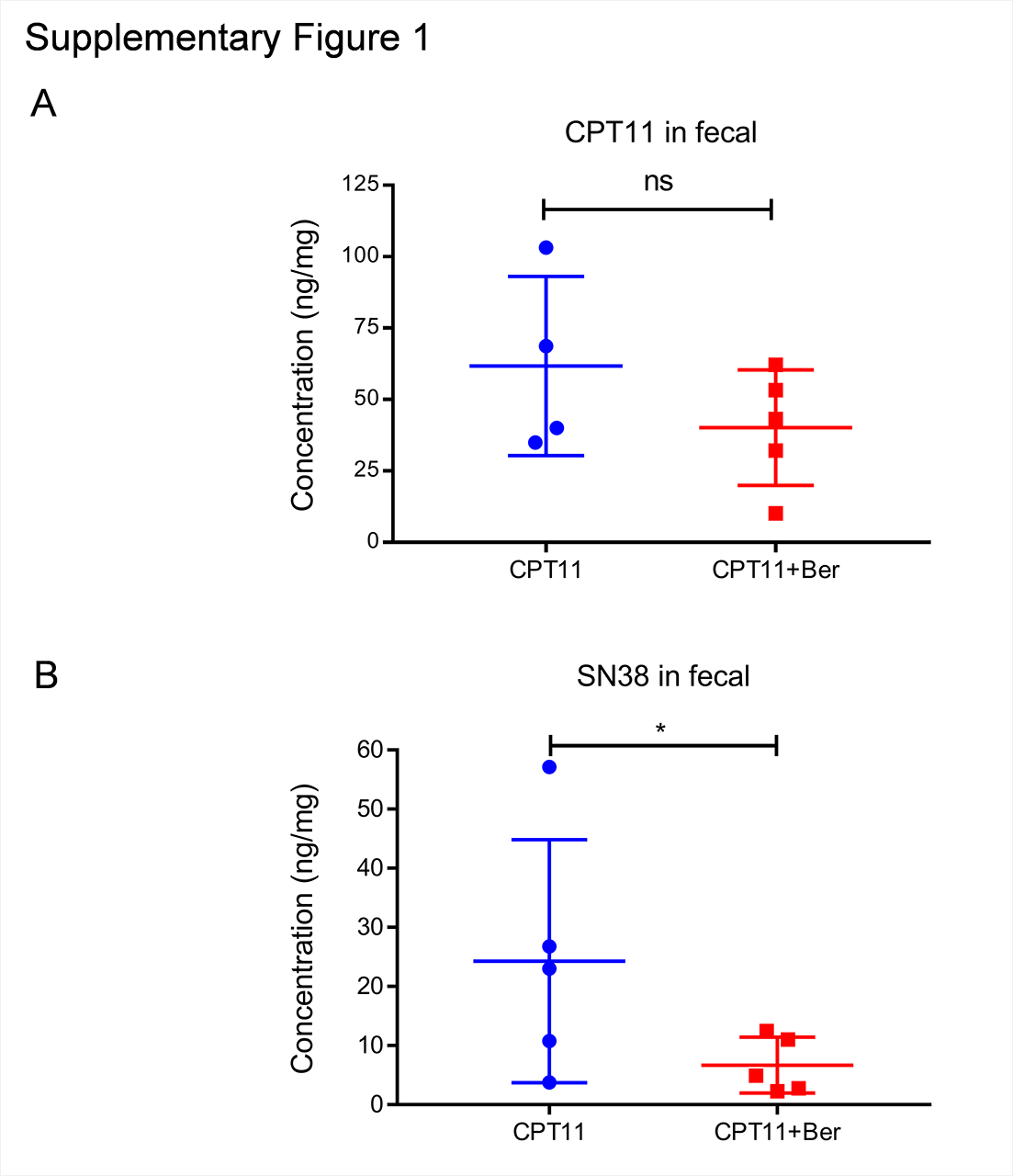

Supplement: Supplementary file 2 [file Image1.TIF]
