# Supplementary material for: Berberine Improves Irinotecan-Induced Intestinal Mucositis Without Impairing the Anti-colorectal Cancer Efficacy of Irinotecan by Inhibiting Bacterial β-glucuronidase
Source: Front Pharmacol. 2021 Nov 2;12:774560. doi: 10.3389/fphar.2021.774560 (PMC8593678; doi:10.3389/fphar.2021.774560)
Supplement: Supplementary file 3 [file DataSheet1.docx]

**Berberine improves irinotecan-induced intestinal mucositis without impairing the anti-colorectal cancer efficacy** **of irinotecan by inhibiting bacterial β-glucuronidase**

This file includes: Supplemental Methods and Figure legend.

**Supplemental Methods**

**Determination of CPT11 and SN38 concentrations by LC-MS/MS**

The analytes, CPT11, SN38 (C_22_H_20_N_2_O_5_, CAS: 86639-52-3; molecular weight: 392.40) were quantified using validated LC-MS method with few modifications (Guan et al., 2017). All fecal samples (50 mg per sample) were homogenized by vortex mixing in 1 mL acetone. All fecal samples at 24h of last dosing CPT11 were collected. After centrifugation for 5 min at 18000 g, 500 μL supernatant was collected to another clean centrifugal tube, 30 ℃ in just to blow to nearly dry nitrogen flow. Then, all samples were redissolved in methanol (500 μL) containing internal standard (voriconazole, 100 ng/ml) (C_16_H_14_F_3_N_5_O, CAS: 137234-62-9; molecular weight: 349.31). The LC conditions were: Agilent® 1100 LC/MS system. Analysis was carried out using Acquity UPLC® HSS T3 (2.1 × 100 mm Column, Waters, Ireland). Mobile phase A (0.1% formic acid in water) and mobile phase B (100% acetonitrile) were operated with a gradient elution at a flow rate of 0.4 ml/min as follows: 30% B→75% B (0–0.5 min), 75% B→80% B (0.5–6 min), 80% B→90% B (6–7 min), 30% B (7.01–10 min). The column temperature were 60 °C and sample temperature were or 4 °C. The injection volume was 10 μL. The MS conditions were: API 5500 Qtrap triple quadrupole mass spectrophotometer (AB Sciex, USA) equipped with a Turbospray TM source. The system was operated in positive electrospray ionization (ESI) and multiple reactions monitoring (MRM) scan mode.

**The antitumor study of berberine *in vivo***

Healthy male 6-week-old BALB/c mice (20-22 g) were purchased from the Shanghai Laboratory Animal Center. All animals were handled according to the principles of the declaration recommendations of the Animal Experimentation Ethics Committee at Shanghai University of Traditional Chinese Medicine (PZSHUTCM200911012). CT26 colon cancer cells (1×10^6^ in 100 μL of PBS) were harvested and subcutaneously injected into the right flank of mice. Three days afterwards, all the tumor-bearing animals were divided into the following groups (n = 6 mice per group), control group, and berberine group. At the end of experiment, all the mice were euthanized under anesthesia and the tumors were removed and weighted. Tumor volume was determined by measuring length (L, mm) and width (W, mm) to calculate volume (V = 0.5×L×W^2^).

**Supplemental Figure legend**

Supplementary figure 1. Concentrations of CPT11 and SN38 in fecal at 24h of last dosing berberine. Data are presented as means ± SD (n=4-5). *p < 0.05 vs. the CPT11 group.

Supplementary figure 2. Berberine inhibited tumor growth in colon cancer xenograft model. (A) Ber (50mg/kg) significantly inhibited tumor growth. (B) Tumor weight were measured after the sacrifice of animal. (C) Tumor volume were measured during the test period. (D) The mice body weight changes were monitored throughout the study. Data are presented as means ± SD (n=5). *p < 0.05, **p < 0.01, ***p < 0.001 vs. the Control group.

**REFERENCES**

Guan, H. Y. and P. F. Li, et al. (2017). Shengjiang Xiexin Decoction Alters Pharmacokinetics of Irinotecan by Regulating Metabolic Enzymes and Transporters: A Multi-Target Therapy for Alleviating the Gastrointestinal Toxicity. Cancer Chemother Pharmacol **8**: 769.
